# Immune Dysregulation in Branched Chain Organic Acidemias

**DOI:** 10.1002/jimd.70203

**Published:** 2026-06-30

**Authors:** Abdul L. Shakerdi, Jack Drda, Justin A. Dutta, Jerry Vockley

**Affiliations:** ^1^ Department of Pediatrics University of Pittsburgh School of Medicine Pittsburgh Pennsylvania USA; ^2^ School of Medicine, Trinity College Dublin Dublin 2 Ireland; ^3^ Department of Human Genetics School of Public Health, University of Pittsburgh Pittsburgh Pennsylvania USA

**Keywords:** cytopenia, immune dysregulation, isovaleric acidemia, methylmalonic acidemia, organic acidemias, propionic acidemia

## Abstract

Organic acidemias (OAs) are a group of inherited disorders, most commonly caused by defects in mitochondrial enzymes involved in amino acid and fatty acid metabolism. While they characteristically present with metabolic and neurological crises, growing evidence reveals a significant burden of chronic immune dysregulation in some disorders and patients. This review provides a synthesis of clinical and mechanistic evidence discussing immune dysregulation in OAs. Cytopenia can occur in OAs and predispose patients to recurrent and severe infections. Adaptive immune deficits, such as hypogammaglobulinemia, reduced B and T cell populations, and impaired vaccine‐specific antibody responses, including to diphtheria and tetanus in MSUD and to the inactivated COVID‐19 vaccine in propionic acidemia, have also been reported. Additionally, some case series note hyperinflammatory conditions, such as hemophagocytic lymphohistiocytosis. Mechanistic studies indicate that accumulated metabolites disrupt innate and adaptive hematopoietic progenitor function, mitochondrial homeostasis, and inflammatory signaling. Emerging therapeutic avenues, such as gene and mRNA‐based therapies, hold the potential to improve or normalize the biochemical phenotype in OAs. While their impact on immune abnormalities remains largely unexplored, future clinical trials offer an opportunity to systematically assess potential effects on immune parameters. OAs are increasingly recognized as disorders with intrinsic immune dysregulation, extending beyond their well‐characterized metabolic and neurological manifestations. Future clinical trials will benefit from including immunological endpoints to evaluate immunological recovery for novel therapies.

## 1 Introduction

Organic acidemias (OAs) are a heterogeneous group of inborn errors of metabolism resulting from defects in multiple enzymes, many of them involved in the catabolism of amino acids, odd‐chain fatty acids, and other metabolic intermediates [1]. This catabolic failure results in the toxic accumulation of organic acids, with metabolic acidosis, with or without ketosis and hyperammonemia [2, 3]. Although significant morbidity and mortality are associated with metabolic crises during catabolic stress, emerging evidence indicates that the neurological, immunological, and hematological systems can also be affected [4, 5]. Despite the frequency with which patients with OAs experience recurrent infections and immune abnormalities, these features remain poorly characterized relative to the metabolic and neurological aspects of disease. A brief overview of the biochemistry of each disorder is therefore provided, as the nature of the accumulating metabolites is central to understanding the immune mechanisms explored in this review.

Propionic acidemia (PA) (OMIM #606054) is caused by a deficiency in the activity of propionyl‐CoA carboxylase (PPA) (EC 6.4.1.3). PPA is a biotin‐dependent enzyme that catalyzes the conversion of propionyl‐CoA into d‐methylmalonyl‐CoA [6]. Deleterious variants in either the *PCCA* (OMIM #232000) or *PCCB* (OMIM #232050) gene lead to the accumulation of propionic acid and other toxic metabolites [7]. PA presents with episodes of metabolic acidosis, hyperammonemia, lethargy, and progressive neurological impairment and cardiomyopathy [8]. Methylmalonic acidemia (MMA) (OMIM #251000) arises due to the impaired conversion of methylmalonyl‐CoA to succinyl‐CoA. This key anaplerotic reaction is catalyzed by methylmalonyl‐CoA mutase (EC 5.4.99.2), encoded by *MUT* (OMIM #609058), and requires adenosylcobalamin as a cofactor [9]. MMA can be caused by mutations in *MUT*, or in genes encoding enzymes involved in the synthesis and transport proteins, such as *MMAB*, *MMADHC*, *MMAA*, and *LMBRD1* [10]. MMA presents with a broad clinical spectrum, including failure to thrive, episodes of metabolic decompensation similar to PA, chronic kidney disease, and developmental delay [11]. Elevated propionylcarnitine (C3) detected on newborn screening is a common early indicator of both PA and MMA [12].

Isovaleric acidemia (IVA) (OMIM #243500) occurs due to pathogenic variants in *IVD* (OMIM #607036), which encodes the flavoenzyme isovaleryl‐CoA dehydrogenase (EC 1.3.8.4). The enzymatic block leads to the accumulation of isovaleric acid and multiple alternative metabolites, producing a distinctive “sweaty feet” odor. It is characterized by episodes of acute metabolic crises with vomiting, lethargy, neutropenia, and thrombocytopenia [13, 14].

Maple syrup urine disease (MSUD) (OMIM #248600) is caused by defects in the branched‐chain α‐ketoacid dehydrogenase (BCKDH) complex (EC 1.2.4.4), responsible for the oxidative decarboxylation of leucine, isoleucine, and valine [15]. Pathogenic variants in any of the genes encoding subunits of this complex (*BCKDHA*, *BCKDHB*, or *DBT*) (OMIM #608348, OMIM #248611, OMIM #248610) result in the accumulation of branched‐chain amino acids and their ketoacids. MSUD typically presents with encephalopathy, dystonia, and a distinct maple syrup odor in urine [15].

3‐Methylcrotonyl‐CoA carboxylase (3‐MCC) deficiency OMIM #210200 results from mutations in either *MCCC1* (OMIM #609010) or *MCCC2* (OMIM #609014), which disrupt leucine catabolism. This disorder is often identified via newborn screening originally with variable clinical penetrance, but now shown to be asymptomatic in most individuals identified by newborn screening [16, 17].

Other OAs arise from deficiencies in all of the biotin‐dependent carboxylases. Multiple carboxylase deficiency includes two distinct disorders, holocarboxylase synthetase deficiency and biotinidase deficiency [18, 19]. These disorders impair the function of multiple enzymes, including propionyl‐CoA carboxylase, 3‐methylcrotonyl‐CoA carboxylase, and pyruvate carboxylase [20], leading to metabolic acidosis, skin rash, hypotonia, alopecia, and developmental regression [21, 22]. Table 1 summarizes the enzyme deficiency, accumulating metabolites, clinical features, and inheritance pattern in classical OAs.

**TABLE 1:** Overview of organic acidemias due to defects in branched chain amino acid metabolism.

Disorder	Enzyme deficiency	Main accumulating metabolites	Primary clinical features	Inheritance
Propionic acidemia	Propionyl‐CoA carboxylase	Propionic acid, 3‐hydroxypropionate, propionylcarnitine (C3).	Metabolic acidosis, hyperammonemia, neurologic impairment	Autosomal recessive
Methylmalonic acidemia (MMA)	Methylmalonyl‐CoA mutase or cobalamin metabolism enzymes	Methylmalonic acid, C3, C3/C2 ratio.	Failure to thrive, kidney disease, developmental delay	Autosomal recessive
Isovaleric acidemia (IVA)	Isovaleryl‐CoA dehydrogenase	Isovaleric acid, isovalerylcarnitine (C5).	Sweaty feet odor, vomiting, neutropenia	Autosomal recessive
Maple syrup urine disease (MSUD)	BCKDH complex	Branched‐chain amino/keto acids.	Encephalopathy, dystonia, maple syrup odor	Autosomal recessive
3‐MCC Deficiency	3‐Methylcrotonyl‐CoA carboxylase	3‐MCC, 3‐hydroxyisovaleric acid, 3‐hydroxyisovalerylcarnitine (C5‐OH).	Often asymptomatic, variable	Autosomal recessive
Multiple carboxylase deficiency (holocarboxylase synthetase deficiency)	Holocarboxylase synthetase	3‐Hydroxyisovaleric acid, 3‐methylcrotonylglycine, lactic acid, propionic acid, 3‐hydroxypropionic acid, C3, C5‐OH.	Neonatal onset with metabolic acidosis, lethargy, hypotonia, seizures, vomiting, rash, alopecia.	Autosomal recessive
Multiple carboxylase deficiency (biotinidase deficiency)	Biotinidase	3‐Hydroxyisovaleric acid, 3‐methylcrotonylglycine, lactic acid, 3‐hydroxypropionic acid, C3, C5‐OH.	Later onset with hypotonia, seizures, developmental delay, ataxia, skin rash, alopecia, hearing, vision problems	Autosomal recessive

Recurrent infections and immune abnormalities represent an increasingly recognized source of morbidity in individuals with OAs. However, their mechanisms and clinical implications remain poorly understood. Across several OA subtypes, studies have reported increased infection rates beginning in early infancy, often necessitating hospitalization and intensive care [23, 24]. These are often accompanied by hematologic abnormalities involving both innate and adaptive immunity, including reduced lymphocyte subsets, hypogammaglobulinemia [24, 25], and hyperinflammatory syndromes such as hemophagocytic lymphohistiocytosis (HLH) [26]. A critical unresolved question is whether these immunological aberrations represent a core component of OA pathophysiology that can persist even during periods of metabolic stability, or whether they are secondary phenomena, arising from catabolic stress, nutritional compromise, or systemic infections. For example, severe bacterial/viral infections can deplete marrow progenitor cells and prolong cellular recovery [27, 28]. This distinction has important implications for clinical management, including decisions regarding prophylactic interventions and long‐term surveillance.

Existing evidence of immune dysregulation in OA remains somewhat fragmented, consisting mainly of case reports and small cohort studies. This manuscript is framed as a hypothesis‐generating synthesis that integrates clinical observations with mechanistic data to propose a conceptual model of immune dysregulation in OAs and to identify key gaps for prospective research. The evidence summarized in this review was obtained through targeted PubMed and Google Scholar searches using terms related to the OAs and immune dysfunction. Given the narrative nature, no strict inclusion or exclusion criteria were set, nor was a specific timeline for publication dates. Quantitative analysis was limited to the descriptive heatmap of immune cell expression of OA‐related genes.

## 2 Immune Dysregulation in OAs

Cytopenia is one of the most common hematologic problems in OAs. In a seminal report, an infant with PA presented with life‐threatening pancytopenia during a neonatal crisis [29]. Kelleher et al. subsequently described two infants with IVA who developed severe pancytopenia in the neonatal period. In their cases, absolute neutrophil counts dropped near zero (nadir ~26–150/μL) and platelets fell to < 1000/μL [14]. Rapid supportive care, including prompt transfusions of blood products, was lifesaving in these reports. Following the pancytopenic crisis, normal development was noted in both patients with no recurrent cytopenia once metabolic control was achieved [14]. Contemporary cohort studies indicate that cytopenia remains a frequent manifestation in both acute and stable phases of OAs. Another evaluated 31 children with various OAs in the nonacidotic phase and found neutropenia in 22.6% of all patients, and notably in 42.6% of those under the age of 3 [30]. Neutropenia was observed in some patients with MMA and in the single patient with PA, whereas no cases of neutropenia were identified among patients with IVA in this cohort. Nine of 11 Mexican patients with PA or MMA (82%) were reported to have some blood count abnormality even during routine follow‐up. Bicytopenia (anemia with one other cytopenia) was the most common finding (in 4 of 11 patients), followed by isolated anemia (3/11). One patient each had lymphopenia or full pancytopenia [31]. These data highlight that cytopenia is not an uncommon but rather a pervasive feature of OAs, especially in infancy. The cytopenia can be persistent or recurrent, and likely correlates with patients' susceptibility to infections. For instance, in a retrospective cohort of 38 Saudi Arabian children with PA, 80% experienced infections during their course, in some cases involving opportunistic and healthcare‐associated pathogens such as *Candida species*, *Pseudomonas aeruginosa* , *Enterobacter cloacae* , *Morganella morganii* , and *Serratia* [32]. Evaluating the pathogen spectrum provides mechanistic clues as to which immune compartments are impaired, as different organisms preferentially exploit specific defense deficits. Invasive *Candida* infections classically point to impaired antifungal innate immunity, especially neutrophil‐mediated killing pathways such as nicotinamide adenine dinucleotide phosphate (NADPH) oxidase‐dependent oxidative burst, as well as disruptions in CARD9‐mediated fungal pattern–recognition signaling, and, in some contexts, compromised T‐cell/Th17‐dependent mucosal defenses [33, 34, 35]. Similarly, *Pseudomonas*, *Enterobacter*, and *Serratia* are often associated with neutropenia or phagocyte dysfunction [36, 37, 38]. Further research relating pathogen susceptibility patterns to specific immunological pathways, such as NADPH oxidase‐dependent phagocyte function or CARD9 signaling, could help better define the basis of immune dysregulation in OA.

In one case study, a 2‐month‐old infant with PA developed secondary HLH during a metabolic decompensation. HLH is an extreme inflammatory syndrome characterized by uncontrolled activation of macrophages and lymphocytes [39]. The infant presented with fever, splenomegaly, pancytopenia, hyperserotonemia, hypofibrinogenemia, and bone marrow hemophagocytes [40], fulfilling HLH‐2004 criteria [41]. Extensive evaluation ruled out primary HLH gene mutations. Instead, the HLH was thought to be precipitated by the metabolic crisis [40]. Additional support for HLH as a serious complication in propionate metabolism defects comes from a case series of three children, two with PA and one with MMA, who fulfilled HLH‐2004 criteria during metabolic crises. While all had cytopenia and bone marrow hemophagocytes, two required plasma exchange, and one succumbed to multiorgan failure despite HLH‐targeted therapy [26]. These cases underscore that OAs can also precipitate hyperinflammatory syndromes in addition to immunodeficiency.

Beyond marrow suppression, OAs have been linked to deficiencies in both the adaptive and humoral immune systems. In one report, a PA patient with persistently low IgG (250 mg/dL) suffered recurrent sinopulmonary infections and required immunoglobulin replacement therapy [42]. After regular IVIG infusions, IgG levels normalized, but the patient later developed neutropenia. Another case series reported that 2 of 4 MMA patients had marginally low IgG levels, and one patient lacked protective antibody titers to rubella. Two other patients in that series had elevated IgE levels [43]. Concomitant parathyroid hormone resistance, along with B‐lymphopenia, has been described in one case, both of which normalized following hemodialysis, dietary intervention, and carnitine supplementation [44]. Given the association of PA with humoral immunity deficits and the recommendation of routine childhood vaccination, a controlled cohort study in Austria investigated the specific IgG response and avidity maturation following measles, mumps, rubella, and diphtheria/tetanus vaccinations in 10 PA patients. While patients with PA generally showed lower average vaccine‐specific IgG concentrations compared to healthy controls, these differences were not statistically significant. Overall, PA patients demonstrated protective antibody levels and adequate avidity responses for most antigens tested [45]. In a case study, a patient receiving the inactivated Sinovac‐CoronaVac COVID‐19 vaccine became seropositive after the second dose, with a rise in neutralizing antibodies, but neutralizing activity remained below the positive threshold, indicating a weak vaccine‐induced immune response [46]. Findings on the vaccine response in PA and other OAs remain scarce and inconclusive, but some evidence suggests a reduction in the ability to mount appropriate neutralizing antibody levels.

A recent cross‐sectional observational study with healthy controls from Turkey [24] evaluated both cellular and humoral immunity in 33 children with MMA, PA, and IVA. During infection‐free periods, patients displayed statistically significant deficits in naïve helper T‐cells and recent thymic emigrants, as well as in memory B‐cell subsets and IgG levels. More than half the cohort had histories of sepsis, and over a third had been admitted to the pediatric intensive care unit, underscoring that adaptive immune dysregulation in OAs might not be transient or isolated to metabolic decompensation, but rather a potentially persistent component of the disease phenotype. In this cohort, diagnoses were heavily skewed toward PA and MMA (21 MMA, 10 PA), with only two patients affected by IVA, and immunologic outcomes were analyzed for the pooled OA group rather than by diagnosis, precluding any statistically meaningful IVA‐PA/MMA comparisons. Although neutropenia was the only immune‐related parameter reported descriptively by diagnosis, occurring in PA (*n* = 4) and MMA (*n* = 3), the absence of neutropenia among the two IVA patients cannot be interpreted as evidence of a different immune phenotype, given the extremely limited sample size. In contrast, other immune abnormalities observed in the study, including reduced IgG levels and alterations in T‐ and B‐cell subsets, were not stratified by diagnosis.

More granular immune phenotyping in 30 Egyptian children with PA or MMA revealed that while serum immunoglobulins were normal in all patients, 56.7% had low absolute CD19+ B‐cell counts, 46.7% had low absolute CD3+ T‐cell counts, 30% had low absolute CD4+ counts, and 26.7% had low absolute CD8+ or CD56+ (NK) counts. Notably, these abnormalities did not differ significantly between metabolically controlled and poorly controlled patients [25]. In contrast, findings from a Mexican cohort revealed that hypogammaglobulinemia, especially of class G, was present in both compensated and decompensated patients with propionate defects independent of nutritional status. IgG levels were significantly lower during decompensated states, yet over half of the patients in stable condition also showed subnormal IgG levels [47]. Collectively, these findings suggest that adaptive immune dysregulation, characterized by persistent lymphocyte abnormalities and, in some cases, immunoglobulin deficiency, is a core feature of OAs. These data suggest that both PA and MMA consistently have adaptive immune defects, including reductions in naïve CD4 T‐cells and recent thymic emigrants that have been specifically associated with sepsis history in this cohort [24], whereas IVA appears to be limited to acute‐crisis cytopenia. Additional disorder‐stratified studies are needed to draw firm conclusions about the nature and severity of adaptive immune involvement across individual OA subtypes. The hypogammaglobulinemia, B‐cell lymphopenia, and reduced memory B‐cell subsets seen in OA patients closely resemble common variable immunodeficiency (CVID), and secondary causes, including inborn errors of metabolism, should be ruled out before a primary CVID diagnosis is made [48]. At least some of these immune abnormalities appear to improve with better metabolic control [44], suggesting that toxic metabolite accumulation drives them rather than a primary lymphocyte defect.

Evaluation of the complement system and isohemagglutinin titers in a non‐acidotic cohort of thirty‐one OA patients revealed subtle but noteworthy alterations. Two patients had low IgM levels, and another two had diminished isohemagglutinin titers. Additionally, anti‐tetanus and anti‐diphtheria IgG levels were low in two MSUD patients, despite full vaccination. Interestingly, elevated complement proteins (C3, C4, and CH50) were observed in approximately one‐third of subjects [49]. This could reflect chronic low‐grade immune activation or compensatory responses to prior metabolic crises. A detailed summary of individual case reports and small patient series is provided in Table 2, while findings from larger cohort and cross‐sectional studies are summarized in Table 3.

**TABLE 2:** Summary of immune findings from individual cases and small series.

References	Disorder(s) assessed	Sample size (*n*)	Age (median/range)	Presentation	Cytopenias reported	Other immune findings	Key interventions/outcomes
Sweetman & Nyhan, 1980 [29]	PA	1	Neonatal	Metabolic crisis	Pancytopenia	—	Protein‐restricted diet; RBC transfusions. Blood counts normalized by day 9; height 3rd percentile, weight 25th percentile, speaking in sentences, riding tricycle.
Kelleher et al., 1980 [14]	IVA	2	Neonatal	Patient 1: Tachypnoea, poor feeding, lethargy, vomiting, dehydration and sweaty sock odor. Patient 2: Poor feeding, lethargy, hypothermia, unresponsiveness, and sweaty sock odor.	Pancytopenia	—	Glycine therapy, low‐leucine formula, and RBC/platelet transfusions. Pancytopenia resolved in both; observed at 24 and 17 months with normal hematologic values and appropriate development.
Medina‐Torres et al., 2021 [31]	PA, MMA	11	11 months to 11 years	Clinically stable	Bicytopenia (36%), anemia (27%)	CD4+ lymphopenia, low CD4/CD8 ratio.	Not specified
Land & Roberts, Abstract [43]	PA	1	Not reported in abstract	History of chronic sinus and urinary tract infections	Neutropenia	Low IgG, neutropenia	IVIG, G‐CSF; ANC rose from 300 to 10 600
Griffin et al., 1996 [45]	PA	1	7 weeks	Tachypnoea, dehydration, lethargy, poor feeding, persistent vomiting, limited weight gain. Labs showed metabolic acidosis and severe hyperammonemia.	Anemia, neutropenia	B cell lymphopenia, hypogammaglobulinemia	Hemodialysis, IV calcium for PTH resistance, carnitine, and dietary restriction. Resolution of hypocalcemia, B cell lymphopenia, and hypogammaglobulinemia. At 30 months: normal growth, moderate developmental delay, no recurrence of immune or severe crises.

**TABLE 3:** Immune abnormalities from larger cohort and cross‐sectional studies.

References	Study design	Disorder(s) assessed	Sample size (*n*)	Age (mean, median, or range)	Phase of disease	Key immune findings	Cytopenias reported	Infectious history
Alaei et al., 2017 [30]	Cross‐sectional	PA, MMA, IVA, MSUD	31	Mean: 49 ± 37 months	Nonacidotic	Neutrophil function largely normal on NBT (93.5%)	Neutropenia (22.6% overall), more common in < 3 years (42.6%).	Recurrent infections in 41.9%; hospital readmission in 58%
Al Essa et al., 1998 [32]	Retrospective cohort	PA	38	No exact median or range, at the time of analysis ~85% were older than 1 year.	Mixed course.	3 patients studied between attacks: severe CD4 deficiency, moderate CD8 deficiency, reversed CD4/CD8 ratio, hypogammaglobulinemia, and reduced lymphocyte blastogenesis (1/3).	Not systematically quantified in this series.	80% of patients experienced infections. Common sites were respiratory, gastrointestinal, and bloodstream. Pathogens included both typical bacteria ( *S. aureus* , *S. pneumoniae* ) and opportunists (Candida, Pseudomonas), plus viral and enteric pathogens.
Altun et al., 2022 [24]	Cross‐sectional observational	PA, MMA, IVA	33 OA and 32 healthy controls	Range: 0.44–15.1 years (Mean 5.89 ± 4.11 years)	Infection‐free, non‐acidotic, and metabolically controlled at sampling.	Reduced ↓ naïve CD4 T cells and recent thymic emigrants, reduced class‐switched and non‐switched memory B cells; IgG lower vs. controls, MHC II on monocytes higher. NK and total T‐cell counts largely similar to controls.	Neutropenia 7/33 (21.2%), Lymphopenia 1/33 (3%)	88% frequent hospital admissions; 39% PICU admissions; 55% sepsis; 39% with Gram‐negative sepsis; 27% fungal sepsis. Lower naïve T cells associated with sepsis; Gram‐negative sepsis linked to low naïve T helper cells, low class‐switched B cells and low RTEs; neutropenia linked to deep soft‐tissue infections; low IgM linked to LRTIs. 57.6% met ≥ 2 primary immunodeficiency warning signs.
Lopez‐Mejía et al., 2024 [48]	Cross‐sectional/longitudinal event‐based	PA, MMA	20 patients, 33 events	Not specified	Compensated and decompensated	IgG low in both phases; immune dysregulation not nutrition‐dependent	Compensated vs. decompensated: Anemia 0% vs. 16%; leukopenia 38% vs. 28%; neutropenia 13% vs. 24%; lymphopenia 13% vs. 28%; thrombocytopenia 25% vs. 28%; bicytopenia 13% vs. 20%; pancytopenia 0% vs. 12%. Not statistically significant in decompensated vs. compensated, except for higher monocytes in compensated.	48% of decompensation events linked to infection; opportunistic infections common.

## 3 Mechanistic Insights Into Immune Dysregulation in OAs

Understanding the molecular mechanisms underlying immune dysregulation in OAs is crucial for elucidating how metabolic derangements contribute to both immunodeficiency and autoinflammation, thereby potentially informing targeted strategies to mitigate infection risk and prevent immune‐mediated tissue damage. Unlike terminally differentiated somatic tissues, many immune cells retain substantial developmental and functional plasticity [50]. Activation of innate and adaptive immune cells is normally accompanied by profound metabolic reprogramming [51, 52]. As such, metabolic disturbances in OAs might dysregulate immune cell activation states or differentiation trajectories. The following mechanistic studies do not establish causality in human disease but provide biological plausibility for how accumulated metabolites and mitochondrial dysfunction could influence immune function in OAs, serving as a basis for further investigation.

### 3.1 Disruption of Colony Growth and Lymphocyte Proliferation

Hutchinson et al. in 1985 investigated the effects of metabolites that accumulate in branched‐chain amino acid disorders on granulocyte‐macrophage progenitors. Strikingly, propionate and isovalerate profoundly suppressed granulocyte colony growth at pathophysiological concentrations (3–6 mM), whereas methylmalonate had a milder suppressive effect, and the parent branched‐chain amino acids had none [53]. In one study, bone marrow aspirates and biopsies taken from a PA infant were examined through light and electron microscopy, revealing marked trilineage dysmyelopoiesis, hemophagocytes, and numerous multinucleated histiocytes and megakaryocytes [54]. The trilineage dysmyelopoiesis, showing disruption in maturation across erythroid, myeloid, and megakaryocytic lineages, indicates that propionic acid‐associated metabolites can broadly impair hematopoiesis, rather than selectively targeting one cell lineage. To explore the pathophysiologic role of PA‐associated metabolites, colony‐forming assays were conducted using mouse erythroid (CFU‐E) and granulocyte‐monocyte (CFU‐GM) progenitors, as well as human CFU‐GM. These assays tested the effects of the infant's serum and several organic acids, including propionic acid, tiglic acid, 3‐hydroxypropionate (3‐OH PA), and glycine. The infant's serum significantly inhibited mouse CFU‐E (43%) and nonsignificantly reduced CFU‐GM (32%) but did not affect human CFU‐GM. Buffered propionic acid induced a concentration‐dependent inhibition of both mouse and human colony formation within the pathophysiologic serum range. Importantly, this inhibition was not due to cytotoxicity, as the viability of bone marrow mononuclear cells remained high after incubation with PA. Other metabolites tested had no inhibitory effects, and glycine even enhanced the growth of human CFU‐GM. These findings implicate propionic acid as a reversible inhibitor of hematopoiesis in PA, likely affecting progenitor cell proliferation and maturation. In a patient with MMA, methylmalonic acid inhibited marrow stem cell growth in a concentration‐dependent and reversible fashion, although with lower potency than propionate or isovalerate [55]. The differential colony suppressive potency of each disorder's accumulating metabolites, propionate and isovalerate being more potent granulopoietic inhibitors than methylmalonate [49], likely partly accounts for these clinical differences. Importantly, hematologic abnormalities have not been reported in IVA patients identified on newborn screening who achieve metabolic stability, reinforcing the idea that cytopenia in IVA is primarily a crisis‐driven finding [56].

Beyond progenitor cells, toxic metabolites can also directly dampen mature lymphocyte functions. The immunological impact of branched‐chain ketoacids that accumulate in MSUD has been examined [57]. In healthy human lymphocyte cultures stimulated with a mitogen, the addition of the MSUD metabolites α‐ketoisocaproic (KIC) and α‐keto‐β‐methylvaleric (KMV) acids significantly and concentration‐dependently suppressed lymphocyte proliferation. At 2.5–5 mM, KIC and KMV markedly inhibited lymphocyte proliferation, while α‐hydroxyisovaleric acid required 5 mM to exert a significant effect, suggesting lower potency. In addition, α‐ketoisovaleric (KIV), a metabolite of valine, showed no suppression, and neither did the parent amino acids. Thus, the immunosuppressive activity is specific to certain organic acid metabolites rather than a general property of all branched‐chain amino acid derivatives. The authors reasoned that the elevated BCAA‐derived acids in MSUD could contribute to patients' high susceptibility to infection by inhibiting lymphocyte proliferation and cellular immune competence.

### 3.2 Mitochondrial Perturbation

Mitochondrial dysfunction has been shown to play a role in the pathogenesis of OAs [58]. For instance, in MMA, disruption of anaplerotic input into the TCA cycle impairs mitochondrial energy metabolism [59]. Cells normally rely on phosphatase and tensin homolog‐induced kinase 1 (PINK1)‐Parkin‐mediated mitophagy to clear dysfunctional mitochondria [60]. However, in MMA, failure of this process leads to the accumulation of damaged mitochondria, exacerbating ROS production and cellular injury [61]. It has been shown that excessive ROS and reactive nitrogen species produced in mitochondrial dysfunction can act as signaling molecules and cellular stressors [62]. In addition, ROS influences immune cell function by modulating the activity of various transcription factors such as nuclear factor kappa B (NF‐κB) and hypoxia‐inducible factor 1‐alpha (HIF‐1α) [63, 64]. NF‐κB drives the transcriptional upregulation of IL‐1β and NOD‐like receptor family pyrin domain‐containing 3 (NLRP3) during inflammatory signaling, effectively priming the inflammasome for activation [65].

In MMA, evidence suggests that oxidative stress contributes to immune dysregulation. Children with MMA (cobalamin C subtype) (OMIM #277400, MMACHC) had significantly elevated serum markers of oxidative damage [malondialdehyde and nitric oxide (NO)], as well as pro‐inflammatory cytokines TNF‐α and IL‐6, compared to healthy controls [66]. Concurrently, the major antioxidant enzymes glutathione and superoxide dismutase were found to be decreased in MMA patients. Notably, higher levels of TNF‐α, IL‐6, NO, and malondialdehyde were correlated with worse cognitive performance in MMA, whereas antioxidant levels correlated positively with cognition [66]. This implies that the degree of systemic inflammation and oxidative stress is linked to disease severity. At the molecular level, pro‐inflammatory cytokines such as TNF‐α and IL‐1β have been shown to perturb mitochondrial function and inhibit respiratory enzymes, along with pyruvate dehydrogenase [67, 68, 69]. In MMA‐treated rats, elevated levels of pro‐inflammatory cytokines, such as IL‐1β and TNF‐α, were observed in both blood and brain tissue, alongside increased inducible nitric oxide synthase (iNOS) expression and 3‐nitrotyrosine accumulation in the cerebral cortex [70]. These changes coincided with spatial memory deficits, suggesting a link between neuroinflammation and cognitive impairment. Concurrently, peripheral immune disruption was evident through reduced neutrophil counts and increased mononuclear cells, mirroring the immunosuppressive and dysregulated immune profiles reported in patients with MMA [70].

### 3.3 Fatty Acids as Signaling Molecules

Another potential metabolic‐immune intersection is the activation of specific signaling pathways by metabolites acting as ligands. Short‐chain fatty acids like propionate can bind to G‐protein‐coupled receptors on cells [71, 72]. One study demonstrated that propionic acid increases the expression of free fatty acid receptor FFAR3 (GPR41) on human neural stem cells in autism spectrum disorder and increases the activity of the phosphoinositide 3‐kinase/protein Kinase B (PI3k/Akt) pathway [73]. Propionic acid exposure led to downregulation of phosphatase and tensin homolog (*PTEN*) and robust Akt phosphorylation, promoting cell survival and proliferation in glial cells. In addition, propionic acid induced a nearly fivefold increase in TNF‐α expression in these cells. Pre‐treatment with 3‐hydroxybutyrate, a short‐chain fatty acid receptor antagonist, blocked the effects of propionate on gliosis and neural stem cell proliferation, supporting that propionic acid's action is mediated via GPR41 signaling, although direct agonism was not demonstrated in this study [73]. Additionally, Akt signaling intersects with NF‐κB and other pathways that regulate immune cell function [74]. Akt plays an essential regulatory role in T cells by phosphorylating the Forkhead box O (*FoxO*) transcription factor family, thereby driving their exclusion from the nucleus [75]. *FoxO* regulates the expression of genes such as Kruppel‐like factor 2 (*KLF2*) and IL‐7Rα, involved in naïve T‐cell homing and survival [76]. Thus, fatty acid metabolite‐receptor interactions link metabolic flux to immune gene expression. It is noteworthy that *GPR41* is expressed not only in the nervous system but also on certain leukocytes, such as polymorphonuclear cells [77]. As such, propionate could similarly influence peripheral immune cell behavior via this receptor, although this remains to be studied.

The gut–immune axis is an emerging field that describes the bidirectional communication between the gut microbiota and the immune system, regulating immune function in health and disease [78, 79]. In PA, the circulating propionate pool is best conceptualized as arising from two major sources: endogenous overflow upstream of the enzymatic block and that generated via gut microbial fermentation. This provides a rationale for gut‐targeted therapy, such as oral metronidazole, aimed at reducing the microbial contribution to the circulating propionate pool, thereby limiting excessive SCFA receptor activation. The use of metronidazole in MMA and PA has been shown to reduce urinary excretion of propionate metabolites and plasma propionate levels [80]. In one case report, probiotic supplementation was administered following metronidazole therapy to selectively modulate the gut microbiota by promoting *Bifidobacterium* species that lack metabolic pathways for propionate production, thereby reducing intestinal contribution to propionate burden in PA [81]. Linking mechanistic insights into SCFA signaling with the potential of gut microbiota modulation, focused mechanistic and clinical studies are needed to establish clinical and therapeutic relevance.

### 3.4 Altered Interferon Signaling Dynamics

**FIGURE 1:**
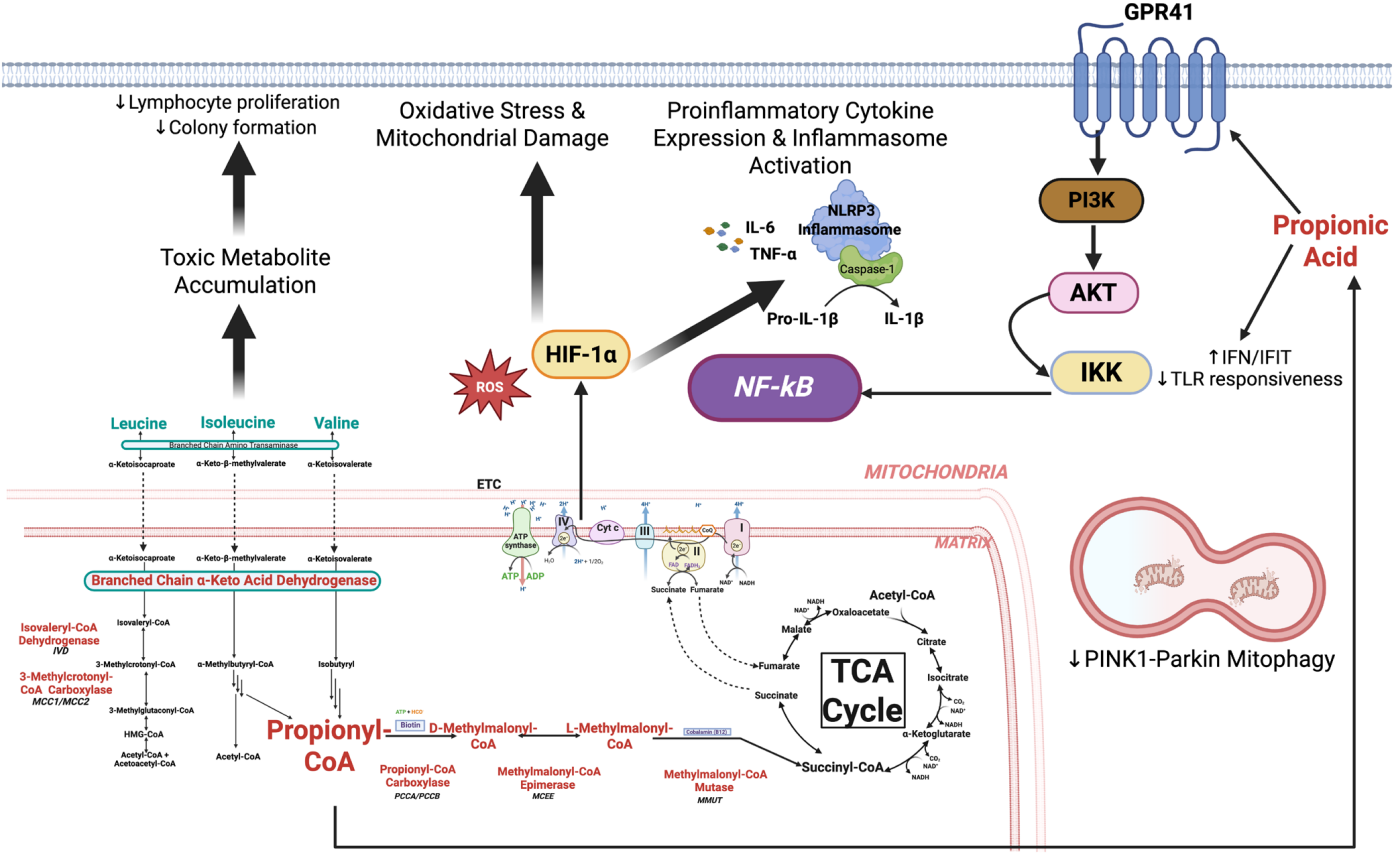
Proposed mechanisms underlying immune dysregulation in organic acidemias. Toxic metabolite accumulation from branched‐chain amino acid catabolism impairs lymphocyte proliferation and colony formation. Mitochondrial dysfunction leads to impaired tricarboxylic acid (TCA) cycle flux, decreased succinyl‐CoA anaplerosis, and accumulation of damaged mitochondria due to defective PINK1‐Parkin mitophagy. These changes promote increased reactive oxygen species (ROS) production and dysregulated inflammatory signaling, including NF‐κB and HIF‐1α pathways. Propionate can function as a signaling molecule via the G‐protein‐coupled receptor GPR41, which stimulates PI3K‐Akt‐IKK pathway activity, further amplifying NF‐κB‐dependent inflammatory responses. Various aspects of this figure are extrapolated from preclinical research in non‐OA contexts, intended for laying the basis for future investigation, and hence should be interpreted with caution. Figure created using BioRender.com.

Aberrations in interferon‐mediated signaling might also contribute to the altered innate immune responsiveness in OAs, though direct evidence is lacking. In human epithelial (A549) and myeloid cells, propionate has been shown to stimulate FFAR2‐Gαq/11 signaling, which induces type I and type III interferon (IFN)‐Janus kinase/signal transducer and activator of transcription (JAK/STAT) activity [82]. This results in the upregulation of interferon‐stimulated genes, including members of the interferon‐induced protein with tetratricopeptide repeats (IFIT) family. In this study, IFIT induction was associated with reduced cellular responsiveness to Toll‐like receptor 1/2 (TLR1/2) and TLR4 agonists, leading to suppressed pro‐inflammatory cytokine, chemokine, and MHC class II expression upon restimulation [82]. Given that these pathways are central to antiviral immunity [83, 84], these findings raise the possibility that propionate‐driven IFN‐IFIT signaling might modulate innate immune responsiveness in OAs and warrant further investigation in disease‐relevant models and patient‐derived immune cells. A summary of the proposed mechanisms leading to immune dysregulation in OAs is provided in Figure 1. It is important to note that the direct link between these mechanisms to outcomes specific to OA remains hypothetical, and this synthesis primarily aims to serve as a basis for future investigation.

**FIGURE 2:**
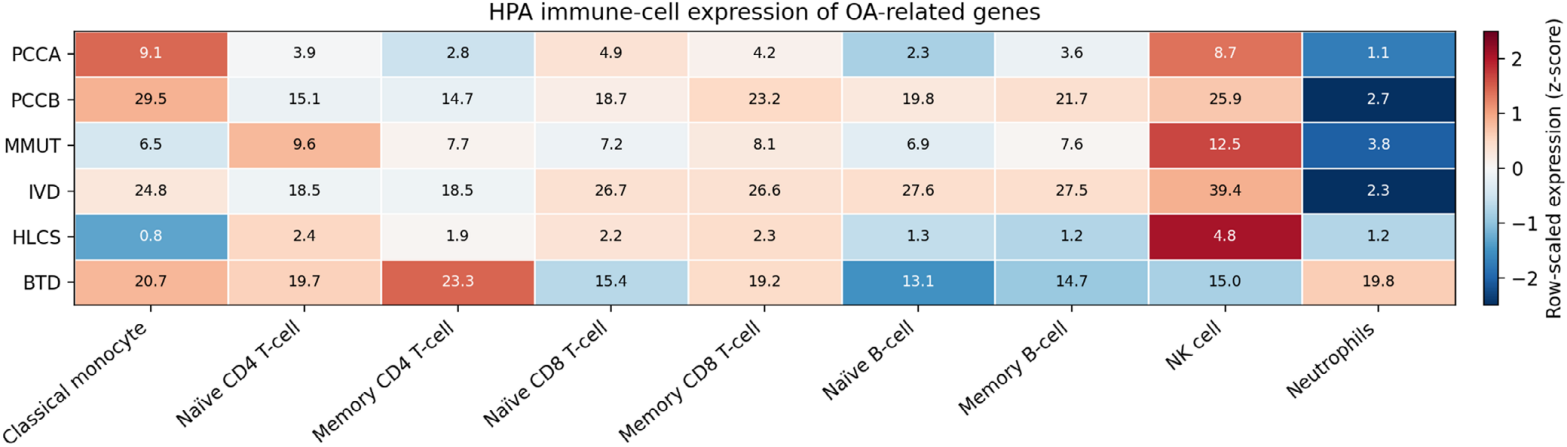
Expression of OA‐associated genes across immune cell populations. Heatmap showing baseline RNA expression of genes implicated in organic acidemias across major innate and adaptive immune cell types, derived from Human Protein Atlas (HPA) bulk RNA‐sequencing data. Expression values are reported as normalized transcripts per million (nTPM). Color intensity reflects row‐scaled expression values calculated from log2‐transformed nTPM to emphasize relative lineage‐specific enrichment, while numeric values within each cell indicate the original nTPM expression levels. The heatmap was generated in Python (Google Colab environment) using Matplotlib, following manual extraction of data from HPA.

Baseline expression of genes implicated in OAs across major innate and adaptive immune cell populations, derived from publicly available Human Protein Atlas (HPA) RNA‐seq data and reported as normalized transcripts per million (nTPM), demonstrates that these genes are expressed in multiple immune lineages (Figure 2). The detection of baseline expression in immune cells supports the biological plausibility of cell‐intrinsic effects of these metabolic defects within the immune compartment, although this alone does not imply functional impairment in OA. Expression is generally higher in lymphoid cells and monocytes than in neutrophils, except in biotinidase deficiency. Importantly, the relatively low expression of several OA‐related genes in neutrophils suggests that the observed neutropenia might not reflect a mature neutrophil–intrinsic enzyme deficiency, but rather indirect mechanisms, such as suppression of hematopoietic progenitors or toxic metabolite effects. Interpretation of these patterns should be made cautiously and primarily as a baseline reference for further research, as transcript abundance alone does not always correlate with protein activity, functional metabolic flux, or immune cell behavior in a disease context. Baseline transcriptional expression levels might also vary depending on immune cell activation or differentiation state.

## 4 Discussion and Future Directions

As presented in this review, there is significant evidence that PA, MMA, and IVA display some degree of immune dysregulation. However, the clinical data presented to date are insufficient to adequately distinguish disease‐specific patterns, especially given that the cohort studies analyzed frequently consist of relatively small samples and are often pooled across diagnoses, thus limiting comparative inference. Nevertheless, some broad conclusions can be drawn, serving as a basis for future investigation. In PA and MMA, immune abnormalities appear to extend beyond transient effects of metabolic decompensation and include recurrent or persistent cytopenias, as well as adaptive immune defects, such as reduced B‐ and T‐cell subsets and hypogammaglobulinemia in a subset of patients. In some cases, hyperinflammatory syndromes, including secondary HLH, occur. These abnormalities have been documented both during metabolic crises and in clinically stable patients, which supports an intrinsic immune vulnerability. In contrast, reported immune involvement in IVA is characterized by acute, often severe neutropenia and thrombocytopenia that occur during metabolic decompensation. Clinical data in MSUD remain limited, though there is some evidence of impaired humoral response following tetanus and diphtheria vaccination. However, experimental studies have shown that branched‐chain ketoacids suppress lymphocyte proliferation, suggesting a plausible mechanism for immune impairment in MSUD and warranting further clinical research. As for 3‐methylcrotonyl‐CoA carboxylase deficiency and multiple carboxylase deficiencies, immune abnormalities have not been well characterized, which could reflect either a true biological difference or simply the rarity of the disorders.

Clinical management of immune dysregulation in OAs requires a proactive and multidisciplinary approach. Cytopenia is common and can be severe in infants. Complete blood counts should be checked regularly, especially during intercurrent illness or decompensation. In cases of severe marrow suppression, timely transfusions of red blood cells and/or platelets can be lifesaving. In addition, G‐CSF can be considered when neutrophil counts are dangerously low. When hypogammaglobulinemia is present, particularly in cases of recurrent infections, immunoglobulin replacement therapy should be considered. Measurement of vaccine‐specific titers, such as for tetanus or MMR, will indicate whether the patient has developed adequate protection, helping to determine if booster vaccines should be given.

Existing OA vaccine data arise from protein‐based or protein‐conjugate antigen vaccines (MMR, diphtheria, tetanus, COVID‐19), which are T‐cell dependent and require intact CD4 T‐cell help for proper maturation and memory responses [45, 46, 49]. While vaccination guidelines for inborn errors of metabolism acknowledge the potential for impaired immune responses in OA patients and recommend additional pneumococcal coverage, systematic immunogenicity data for either antigen type in this population remain scarce [85, 86]. There are no published data on responses to T‐independent polysaccharide antigens such as PPSV23—the standard tool for assessing B‐cell intrinsic antibody capacity in immunodeficiency workups—in OA patients [87]. The naïve CD4 T‐cell depletion and reduced numbers of recent thymic emigrants documented in OA patients [24] likely contribute to the impaired T‐dependent vaccine responses observed in this population, although data remain limited. Whether T‐independent polysaccharide responses are similarly affected remains untested, but given the B‐cell lymphopenia and reduced memory B‐cell subsets documented in OA cohorts, pneumococcal and meningococcal protection might be lower than assumed, and conjugate formulations preferable in this population [24, 25]. Systematic prospective vaccine immunogenicity studies covering both T‐dependent and T‐independent antigens are needed to address these gaps.

Most patients appear to maintain adequate immune memory, so routine vaccinations should be administered on schedule unless clearly contraindicated. For those with signs and markers of systemic inflammation, such as persistent fevers, cytopenia, or organomegaly, secondary HLH should be considered. If suspected, early referral and prompt treatment with immunomodulatory agents will likely be warranted. In some patients, immune function has been shown to improve with metabolic control through diet, carnitine supplementation, and dialysis. A future area of research is whether oral antibiotics or probiotics/prebiotics can ameliorate immune dysregulation in OA by reducing the microbial‐derived organic acid burden and subsequent metabolite signaling, particularly in PA. It is important to note that, as with many rare inherited metabolic disorders, management of immune dysfunction in OA lacks disease‐specific guidelines and remains an open area of discussion.

In the setting of cytokine‐driven hyperinflammation, off‐label use of biologic agents has shown promise. For instance, anakinra, an interleukin‐1 receptor targeting monoclonal antibody, has been successfully used in a child with MMA and multisystem inflammatory syndrome in children (MIS‐C) [88]. Although MIS‐C in this case was not directly attributed to the underlying metabolic disorder and was likely coincidental, the favorable response to IL‐1 blockade provides proof of concept for the potential utility of targeted anti‐cytokine therapy in hyperinflammatory states associated with OAs. In a separate case, a child with MMA presented with concurrent MIS‐C and Kawasaki disease‐like features during dual SARS‐CoV‐2 and *Mycoplasma pneumoniae* co‐infection, with coronary artery dilation, pancytopenia attributed to hematopoietic dysfunction, and a suboptimal response to standard IVIG and glucocorticoid therapy [89]. Kawasaki disease and MIS‐C should be considered in OA patients who present with prolonged fever and signs of systemic inflammation.

Similarly, in GSD‐Ib, infliximab has achieved significant improvement in some patients with refractory Crohn's or Crohn's‐like colitis [90], though outcomes have been variable [91]. In the second case, empagliflozin monotherapy led to clinical remission of severe disease [91]. This suggests that restoring immune function at the metabolic level might obviate the need for prolonged immunosuppressive therapy in certain inflammatory conditions. Lastly, while anti‐cytokine biologics are not currently part of the standard of care for OAs, their efficacy and safety have been established in other metabolic conditions. Mevalonate kinase deficiency (MKD), an inborn error of metabolism characterized by spontaneous, periodic fevers, is treated with anti–IL‐1 biologics to manage its autoinflammatory manifestations [92]. These findings also support the incorporation of cytokine profiling into the work‐up of patients with inflammatory syndromes associated with inherited errors of metabolism to guide targeted therapy.

Gene therapy and mRNA‐based therapy have emerged as promising treatment strategies aimed at correcting the underlying metabolic defects in OAs [93, 94]. Therapies capable of restoring deficient enzyme activity in PA and MMA should improve metabolic control and, secondarily, ameliorate the associated immune dysregulation. Adeno‐associated virus (AAV) tropism is a key practical determinant of whether immune abnormalities can be corrected directly by gene therapy. Preclinical systemic AAV44.9 studies in a PA mouse model demonstrate predominantly hepatic and cardiac expression [93]. Consequently, any improvement in immune function would most plausibly occur indirectly, by reducing the accumulation of toxic metabolites and subsequently correcting downstream cell signaling and progenitor proliferation. It is therefore crucial that immune outcomes are tracked as key secondary endpoints alongside metabolic outcomes in future trials. Early clinical signals in gene and mRNA therapies are encouraging. A first‐in‐human Phase 1/2 trial of mRNA‐3927, encoding the two subunits of propionyl‐CoA carboxylase, for propionic acidemia, has reported a substantial reduction in the frequency of metabolic decompensation events in treated patients. Interim data showed approximately a 70% reduction in the relative risk of metabolic crises with mRNA therapy [95]. For example, gene therapy trials in adenosine deaminase‐deficient severe combined immunodeficiency (ADA‐SCID), which results in the toxic accumulation of purine metabolites and subsequently cytotoxicity to immune cells, have demonstrated improvement in various immunological endpoints, such as lymphocyte subset recovery and immunoglobulin production. While OAs are not primary immunodeficiencies, we suggest that sponsors include exploratory immunological endpoints in their protocols. These could include quantitative measures such as immunoglobulin levels, B/T/NK cell counts, and neutrophil counts, as well as qualitative measures such as infection frequency. A complete cure should ideally normalize immune function and susceptibility to infection, in addition to addressing metabolic disturbances. If that is not achieved, patients would still require adjunct immunological support.

Altogether, the available evidence is consistent with the view that OAs are multisystem disorders in which metabolic perturbations intersect with immune and inflammatory pathways. As discussed, in MMA (cblC/MMACHC), higher circulating pro‐inflammatory cytokine and oxidative stress marker levels have been reported to correlate with worse cognitive performance. In contrast, antioxidants correlated positively with cognition, linking systemic inflammatory and oxidative stress with neurodevelopmental effects. Preclinical work similarly shows that experimental chronic exposure to methylmalonate is associated with increases in inflammatory and nitrosative markers in brain tissue and with spatial memory deficits. Importantly, immune abnormalities could be modifiable with improved metabolic control. In a single reported PA case, B‐cell lymphopenia and hypogammaglobulinemia resolved following aggressive metabolic management (including hemodialysis and dietary intervention), suggesting that correcting metabolic aberrations will normalize at least some immune abnormalities. Collectively, these observations support the view that immune dysregulation in OAs is not solely a transient byproduct of acute crises but rather an ongoing, potentially modifiable component of disease biology that merits consideration in both preclinical research and outcome assessments of novel therapies.

Several limitations should be noted when interpreting the findings of this review, primarily stemming from the limited evidence base available for these rare disorders. While the narrative, rather than systematic, approach enables consideration of a broad range of clinical and experimental evidence, it excludes a prespecified search strategy, formal risk‐of‐bias assessment, or formal statistical analysis. As such, conclusions should be interpreted within these methodological constraints. The underlying evidence base largely consists of small case reports/series and cross‐sectional observational studies with relatively small sample sizes, limiting statistical power and robustness of findings. In addition, many studies aggregate multiple OA diagnoses rather than analyzing disorders individually. This constrains disorder‐specific inference and limits the analysis of some biological and clinical differences across the conditions. The populations in the studies have different cohort characteristics with respect to age, OA disease stage, medical history, and comorbidities, which contributes to heterogeneity and limits cross‐study comparison. The mechanistic hypotheses discussed should be interpreted with caution as they integrate findings from preclinical research and functional biological studies and may not directly recapitulate immune biology in OAs. Another important consideration is that immune outcomes vary widely across studies, ranging from basic hematological laboratory values to detailed functional assays, particularly in mechanistic studies, which could involve different laboratory methodologies and reference ranges. In addition, most available studies provide cross‐sectional snapshots of immune status rather than longitudinal assessments, which limits the characterization of long‐term trajectories of immune function and the cumulative impact of repeated metabolic decompensations. These limitations underscore the need for larger, multicenter, longitudinal studies with standardized immune assessments and detailed clinical phenotyping.

To build on the current mechanistic understanding of immune dysregulation in OAs, several research strategies can be pursued. First, omics‐driven profiling of patient samples across different metabolic states can elucidate immune pathways perturbed by metabolic crises. For example, comparative transcriptomics or proteomics of immune cells obtained during a decompensation versus during stability could identify signatures of immune cell exhaustion, activation, or developmental arrest attributable to metabolite exposure. Similarly, metabolomic profiling of patient plasma could be correlated with immune phenotypes. Recent developments in single‐cell immunometabolism techniques enable the ex vivo metabolic profiling of steady‐state and activated immune cell subsets in human patients and translational mouse models [96, 97]. On the therapeutic side, studies should test preventive immunomodulation in animal models. For instance, administering G‐CSF in PA animal models under metabolic stress to see whether it averts neutropenia and improves infection outcomes, or conversely, whether it precipitates metabolic deterioration. Likewise, immune‐targeted drugs, such as cytokine‐targeting biologics, could be tested in models to assess their effects on both immune parameters and metabolic status.

## 5 Conclusion

OAs are increasingly being recognized as disorders with intrinsic immune dysregulation, extending beyond their well‐characterized metabolic and neurological manifestations. Evidence from clinical cohorts, case reports, and mechanistic studies demonstrates that cytopenia, especially neutropenia, as well as adaptive immune abnormalities such as hypogammaglobulinemia and lymphocyte depletion, are pervasive features even during metabolically stable periods. Accumulated metabolites, such as propionate, methylmalonate, and isovalerate, have been shown to impair hematopoietic progenitor cell proliferation across multiple lineages, disrupt mitochondrial function, and promote oxidative stress and pro‐inflammatory signaling, providing a mechanistic basis for the observed immune abnormalities. While emerging therapies, such as gene‐ and mRNA‐based. treatments, hold promise for metabolic correction, their potential to reverse or ameliorate immune dysregulation remains an open question. Future studies and clinical trials should include immunological endpoints to determine whether restoring metabolic homeostasis also normalizes immune function. Integrating immune monitoring into the care and study of patients with these disorders will be essential to fully understand and address the multisystemic nature of OAs.

## Author Contributions

**Abdul L. Shakerdi:** conceptualization, writing – original draft, visualization. **Jack Drda:** writing – original draft, visualization. **Justin A. Dutta:** writing – reviewing drafts. **Jerry Vockley:** conceptualization, writing – original draft, writing – reviewing drafts.

## Funding

J.V. was funded in part by NIH grant 1U54HD121579‐01.

## Ethics Statement

Ethical approval was not required for this work as it is a narrative review and does not include new studies involving human participants or animals.

## Consent

The authors have nothing to report.

## Conflicts of Interest

The authors declare no conflicts of interest.

## Data Availability

Data sharing not applicable to this article as no datasets were generated or analysed during the current study.
